# Leaf functional traits highlight phenotypic variation of two tree species in the urban environment

**DOI:** 10.3389/fpls.2024.1450723

**Published:** 2024-12-17

**Authors:** Ahram Cho, Nicole Dziedzic, Aria Davis, Cindy Hanson, Jangho Lee, Gabriela C. Nunez-Mir, Miquel A. Gonzalez-Meler

**Affiliations:** ^1^ Department of Biological Sciences, University of Illinois Chicago, Chicago, IL, United States; ^2^ Department of Earth and Environmental Sciences, University of Illinois Chicago, Chicago, IL, United States

**Keywords:** Chicago Metropolitan Region, elemental analysis, gas exchange, land surface temperature, leaf economic spectrum, urban heat island effect, water-usage strategy

## Abstract

Urbanization is transforming landscapes globally, altering environmental conditions that affect ecosystem functioning, particularly in urban areas where trees are crucial for regulating microclimates, improving air quality, and sustaining biodiversity. This study investigates the environmental differences and tree leaf structure and morphology in urban and suburban sites in the Chicago Metropolitan Region. The leaf functional traits of Norway Maple and Little − leaved Linden were studied in three locations in the summer of 2023: an urban park (University of Illinois Chicago, Chicago, IL), a suburban park (Morton Arboretum, Lisle, IL), and a suburban residential site (Lombard, IL). The urban site had higher daytime and nighttime air, and land surface temperatures compared to the suburban sites with significant fluctuations observed across the sites. Cumulative growing degree days, a measure of potential photosynthetically active days, were also higher in the urban park than in the suburban sites between March and August. Norway Maple trees growing in the urban site displayed higher specific leaf area (SLA) and lower leaf dry matter content (LDMC) than in the suburban sites, resulting in thinner leaves. Similarly, Little−leaved Linden trees in the suburban residential site displayed higher SLA and lower LDMC than those in the suburban park. The values of gas exchange traits − namely photosynthetic assimilation, transpiration rates, and stomatal conductance − of Norway Maple were higher at the urban site compared to suburban sites as temperatures increased during the summer. Norway Maple gas exchange values decreased as the growing season progressed, as expected by ontogeny. In contrast, Little−leaved Linden maintained similar leaf gas exchange values throughout the growing season. Both species in the urban site exhibited lower instantaneous water use efficiency and reduced LDMC, suggesting greater water loss in response to elevated temperatures compared to suburban park and residential sites. Comparisons with existing global trait databases emphasize the need for localized data to accurately capture site−specific responses. Although some traits aligned with database values, others deviated significantly, underscoring the importance of comprehensive, site−specific datasets for robust ecosystem modeling and management strategies.

## 1 Introduction

Cities are projected to accommodate 70% of the global population by 2030 (Helletsgruber et al., 2020). Many cities have been experiencing unprecedented growth, which has led to significant environmental degradation (Roy et al., 2012). Urban trees play a vital role in improving urban livability by mitigating urban heat and air pollution, reducing stormwater damage and flooding, and sequestering carbon dioxide (Salmond et al., 2016). Expanding urban parks has been linked to reductions in particular matter diameter ≤ 2.5 µm (PM_2.5_), a fine pollutant known to harm human health, while also providing cooling on hot summer days (Cao et al., 2024).

The diverse environments in which urban trees grow, and their long lifespans make them valuable indicators of plant responses to climate change (Farrell et al., 2015). To understand how urban trees provide benefits and adapt to their unique environment, we can examine their leaf functional traits (LFTs). LFTs, such as specific leaf area (SLA) and photosynthetic rates, are crucial to plant performance and fitness. These traits are defined by their morpho − physio − phenological characteristics and reveal how urban trees respond to environmental stressors, including water availability (Violle et al., 2007). Previous studies have shown divergent responses of urban trees to stressors, with some exhibiting larger SLA and lower leaf dry matter content (LDMC), while others displayed the opposite trend to cope with stressful conditions (Zhu et al., 2020; Salamanca-Fonseca et al., 2023). Plants exhibit diverse water use strategies to mitigate water stress. Some species, known as isohydric plants, prioritize maintaining constant water potential in their leaves by closing stomata and reducing water loss during drought, while anisohydric plants tolerate lower water potentials and keep their stomata open to maintain gas exchange even under drought conditions (Sade et al., 2012). This heterogeneity underscores the importance of considering phenotypic plasticity in trees across varying environmental conditions. Significant foliar changes, including differences in SLA, LDMC, photosynthesis, and transpiration, can occur between species and groups and even within individual trees due to varying ecological conditions across tree canopy positions (Vega et al., 2020).

Urban areas, characterized by extensive buildings and paved surfaces, tend to have higher temperatures than surrounding non−urban areas, a phenomenon known as the urban heat island (UHI) effect (Taha et al., 1988). The surface UHI (SUHI), representing the difference in land surface temperatures (LST) between urban and non−urban surfaces, can be measured by satellite thermal remote sensing data (Zhou et al., 2018). This effect, caused by changes in surface energy balance due to urbanization, results in elevated LST compared to suburban and rural areas (Mutiibwa et al., 2015; Zhou et al., 2018). In urban environments, trees face significantly warmer temperatures, up to 12°C higher than in nearby suburban regions (Oke, 1973; Lahr et al., 2018). These elevated temperatures significantly impact the physiological processes of trees, including photosynthetic assimilation and transpiration rates, and stomatal conductance (*g*_s_), which are all interrelated physiological determinants of plant growth (Urban et al., 2017). Additionally, soil moisture influences gas exchange by affecting *g*_s_; lower soil moisture can lead to reduced stomatal opening, limiting CO_2_ uptake and photosynthetic efficiency, especially under elevated temperatures associated with the UHI effect (Chen et al., 2023).

Chicago’s Metropolitan Region is a high−density residential area with a population exceeding 9.5 million along the shore of Lake Michigan that experiences diverse weather patterns (Wang et al., 2023). Despite the extensive presence of street and residential trees, urban trees in Chicago often face severe stress, especially in street−side locations (Mcpherson et al., 1997; Ballach et al., 1998; Pickett et al., 2001; Salmond et al., 2016). Analyzing leaf structure and biochemical traits in urban trees is a resource−extensive process due to variations in leaf properties throughout the canopy, requiring expensive, specialized equipment and expertise (Burnett et al., 2021). Nonetheless, studying changes in LFTs along the urban − suburban gradient in the Chicago Metropolitan Region can provide insights into plant responses and adaptations to urban stressors under a rapidly changing climate.

Here, we aim to understand how vegetation responds to local environmental conditions, specifically across both urban and suburban settings. We use LFTs, such as morphological traits, gas exchange processes, and the elemental composition of carbon and nitrogen, to investigate the effects of the local environment on two common deciduous tree species, Norway Maple and Little-leaved Linden. This approach will capture variations in how urban trees adapt to the resource availability in urban areas and inform the implications of our leaf−scale understanding of phenotypic plasticity on a plant trait database.

## 2 Materials and methods

### 2.1 Study area and characteristics of sampled species

Norway Maple (*Acer platanoides*) and Little−leaved Linden (*Tilia cordata*) were selected as the study species due to their adaptability to the dry and alkaline soil conditions prevalent in the Chicago Metropolitan Region (Morton Arboretum, 2024). These species constitute a significant portion of the tree population in Chicago, accounting for 11% (Norway Maple) and < 1% (Little−leaved Linden) in the region (Morton Arboretum, 2020a, Morton Arboretum, 2020b; Nowak et al., 2013).

**Figure 1:**
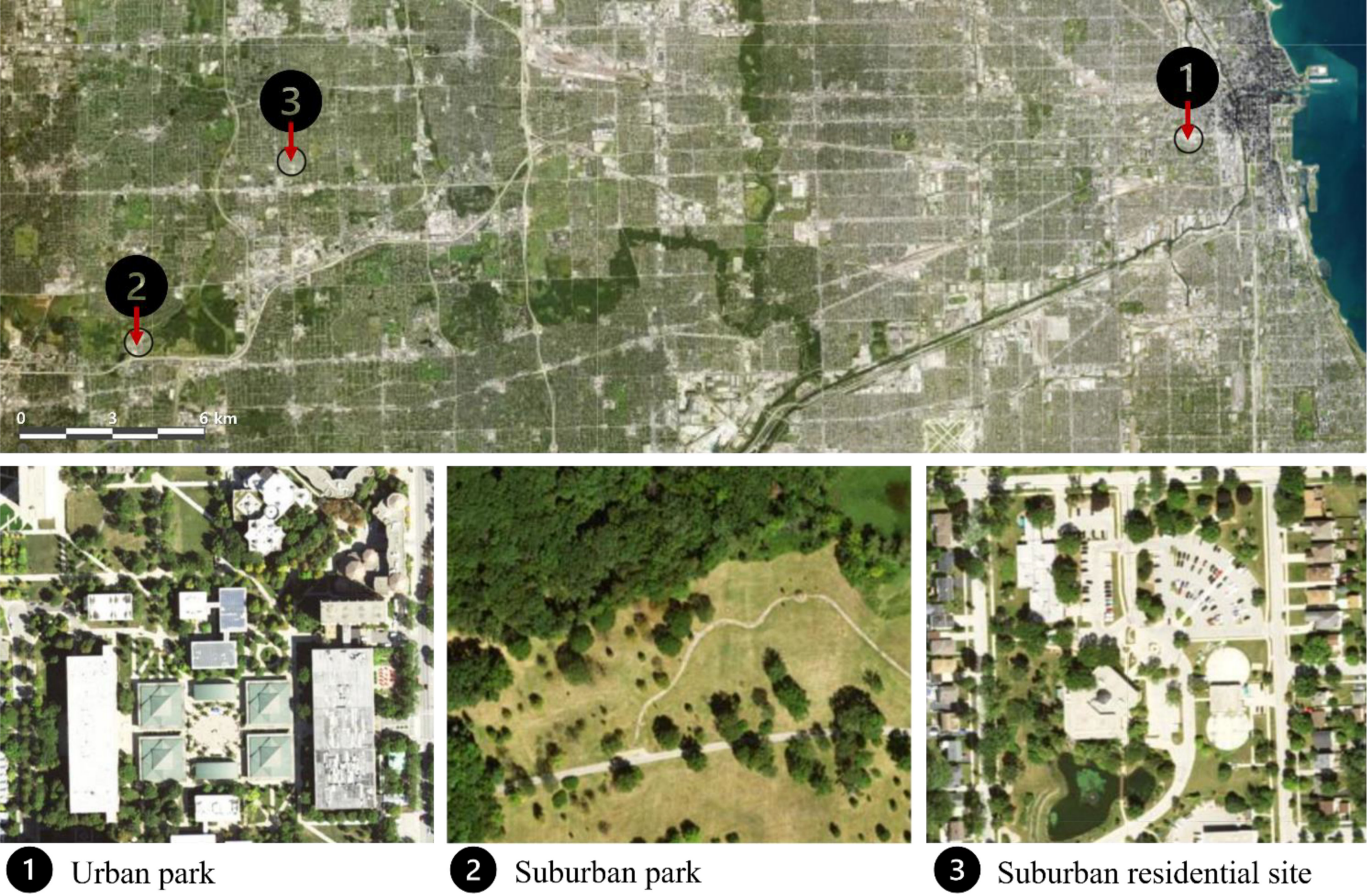
Map showing site locations. Sites 1, 2, and 3 are the urban park (University of Illinois Chicago, Chicago, IL), suburban park (Morton Arboretum, Lisle, IL), and suburban residential sites (Lombard Village Hall, Lombard, IL), respectively. Images in Figure 1 were obtained from NAIP imagery viewed within ArcGIS (www.arcgis.com/home/item.html?id=d1cd1b03d7f246d5a29597f71820ea5d; data accessed Jan 5, 2024). Images have been annotated with identifying information. The scale bar represents 6 km.

Sampling was conducted in the summer of 2023 at three sites: one urban site (urban park) in Chicago, IL, and two suburban sites (suburban park and suburban residential) in Lisle and Lombard, IL, respectively (**Figure 1**). The urban park site, situated at the University of Illinois Chicago (UIC), is a public park (W Vernon Park) with adjacent forest fragments and substantial built infrastructure (paved areas and buildings). The suburban park site is a public park (Morton Arboretum) with various wild and planted tree cultivars growing in an open area. The suburban residential site encompasses a parking area at Lombard Village Hall, with an adjacent greenway less than 6 m away. **Table 1** presents detailed information on the sampling dates, the number of trees measured, the range of diameter at breast height (DBH), and soil moisture levels. The soil moisture was measured as volumetric water content (%) in the top 12 cm of soil within a 1 m radius around each tree using a handheld soil water reflectometer (Hydrosense ΙΙ CS659 12 cm rods, Campbell Scientific, Logan, UT, US). No rainfall was recorded at any site between July 14th and 28th, 2023.

**Table 1:** Sampling date, tree count, diameter at breast height (DBH), and land cover, and soil moisture estimated as volumetric water content (%) of *Acer platanoides* (Norway Maple) and *Tilia cordata* (Little−leaved Linden) trees measured during the 2023 sampling season across three sites in the Chicago Metropolitan Region.

Species	Sites	Sampling Date	N	DBH (cm)	Soil moisture (%)	
					Summer (Jul)	Fall (Aug − Oct)
Norway Maple	Urban park	July 25, October 6	4	36.3 ± 4.0	48.0 ± 5.9	16.3 ± 2.1
	Suburban park	July 27, September 22	3	55.6 ± 4.1	20.1 ± 3.7	29.5 ± 8.0
	Suburban residential site	July 17, September 22	3	40.0 ± 3.7	20.6 ± 2.9	13.7 ± 5.1
Little− leaved Linden	Urban park	July 24, October 6	5	48.2 ± 9.3	68.5 ± 17.2	21.0 ± 5.8
	Suburban park	July 27, September 22	5	23.2 ± 15.7	17.8 ± 5.2	24.1 ± 5.6
	Suburban residential site	July 17, September 22	3	47.6 ± 3.9	22.4 ± 9.4	15.9 ± 12.8

The juvenile period for Norway Maple is approximately 15–20 years (Hackett, 2011), while for Little-leaved Linden, it spans 15–25 years (De Jaegere et al., 2016). The Norway Maple trees sampled were mature and had surpassed the juvenile phase, whereas the Little − leaved Linden trees included both juvenile and mature individuals. Including juvenile trees not only allowed us to assess a broader range of developmental stages but also increased the sample size, providing more robust data for analysis. DBH was assessed at all sites as a tree structural indicator (**Table 1**). Norway Maple trees had an average DBH of 36.3, 55.6, and 40.0 cm in urban park, suburban park, and suburban residential sites, respectively, with variations of 3.7 to 4.1 cm (**Table 1**). Little−leaved Linden trees in the urban park and suburban residential sites had similar average DBH of 48.2 cm and 47.6 cm, respectively, with variations of 3.9 to 9.3 cm. However, the DBH of Little-leaved Linden at suburban park was 23.2 cm with a variation of 15.7 cm.

### 2.2 Land surface temperature and growing degree days

Land surface temperature (LST) measurements for Chicago were obtained from the GOES−16, GOES−17, and GOES−18 satellite constellations (hereafter referred to as GOES). The GOES series can measure LST in the North American region at hourly intervals with a 2 km resolution (Yu and Yu, 2020). For our analysis, we selected the nearest GOES grid point for three locations: an urban park at 41°52’24.2”N, 87°38’53.9”W (University of Illinois Chicago, Chicago, IL), a suburban park at 41°48’44.5”N, 88°03’05.3”W (Lisle, IL), and a suburban residential site at 41°52’02.9”N, 88°00’38.6”W (Lombard, IL). Land cover at these sites was analyzed using Sentinel−2 land cover data with a 10 m resolution. The urban park comprised 77% built-up area and 23% vegetated area. The suburban park included 30% built-up area, 35% tree cover, 11% bare ground, and 24% vegetated area. The suburban residential site consisted of 74% built-up area, 22% vegetated area, and 4% bare ground.

The inherent limitation of satellite measurements is their inability to assess surface properties under cloudy conditions. To address this limitation and facilitate a fair comparison between the sites, we used LST measurements exclusively from cloud−free hours at all sites to ensure that all sites were visible from space. Consequently, some hourly data were excluded from the LST dataset, however, all three sites (urban park, suburban park, and suburban residential site) retained consistent data coverage each day. Given that electron transport dynamics in tree species are particularly active during the growing season (March to October) (Pieruschka et al., 2014), average LST values for this period are presented in **Table 2**. Additionally, weekly LST from 2018 to 2022 are shown in **Supplementary Figure 1**.

**Table 2:** The growing season (April to October) land surface temperature (LST) with standard deviation measured during daytime and nighttime at urban park, suburban park, and suburban residential sites from 2018 to 2022. LST was obtained from GOES−16, GOES−17, and GOES−18 satellite constellations in three locations: an urban park at 41°52’24.2”N, 87°38’53.9”W (University of Illinois Chicago, Chicago, IL), a suburban park at 41°48’44.5”N, 88°03’05.3”W (Lisle, IL), and a suburban residential site at 41°52’02.9”N, 88°00’38.6”W (Lombard, IL).

Time	Site	2018	2019	2020	2021	2022	2018 −2022
Daytime	Urban park	21.3 ± 8.6	20.8 ± 7.4	21.9 ± 7.5	22.9 ± 7.6	22.3 ± 8.6	21.9 ± 8.0
	Suburban park	19.8 ± 8.4	19.9 ± 7.2	20.8 ± 7.0	21.4 ± 6.4	21.0 ± 7.5	20.6 ± 7.4
	Suburban residential	20.7 ± 8.7	20.7 ± 7.6	21.7 ± 7.2	22.3 ± 6.7	21.8 ± 7.9	21.4 ± 7.6
Nighttime	Urban park	14.0 ± 8.4	14.0 ± 7.1	14.4 ± 7.5	15.5 ± 6.7	14.4 ± 8.4	14.5 ± 7.7
	Suburban park	13.2 ± 8.5	13.6 ± 6.8	13.4 ± 7.3	14.5 ± 7.3	14.1 ± 7.3	13.8 ± 7.3
	Suburban residential	13.5 ± 8.5	13.8 ± 6.8	13.5 ± 7.5	14.6 ± 6.7	14.2 ± 7.4	13.9 ± 7.4

Daily ambient temperature (T_a_) records for urban park (Chicago, IL) and suburban park sites (Lisle, IL) from March−November 2023 were obtained from the National Oceanic and Atmospheric Administration (NOAA, 2023). Two weather stations were utilized for the urban and suburban park sites: Chicago Midway Airport at 41°47’24.0” N, 87°44’24.0” W and Lisle Morton Arboretum Station at 41°48’36.0” N, 88°03’36.0” W, respectively. The difference between LST and T_a_ helps us understand the climatic processes and impacts of urbanization in different areas (Do Nascimento et al., 2022). The distance between the weather stations and the LST sites were 11.5 km for the urban park and 0.8 km for the suburban park. Air temperatures in urban sites exhibit greater heterogeneity, and the temperature gradient, which influences UHI statistics, extends over distances of up to 18 km (Van Hove et al., 2015). For analyzing the differences between LST and T_a_, only dates with available LST data were included.

Growing degree days (GDD) are a weather−based indicator that tracks the physiological changes of insects and plants. GDD was calculated for the urban park, suburban park, and suburban residential sites in 2023 using data from three weather stations: the West Loop – Chicago Old St. Pats station at 41°52’48.0”N, 87°38’24.0”W for the urban park (selected due to its more recent data availability in 2023), Lisle Morton Arboretum Station at 41°48’36.0”N, 88°03’36.0”W for the suburban park, and the Downtown station in Lombard at 41°53’24.0”N, 88°01’12.0”W for the suburban residential site. This differs from the station used for T_a_−LST calculations for the urban park. Accumulated GDD from March to November 2023 was determined by summing all daily Ta values exceeding the minimum development threshold, using a baseline temperature of 5°C for cool-season plants.

### 2.3 Leaf morphological traits

Tree shoots were obtained in June 2023 by cutting branches longer than 50 cm from the southern side of the crown at an intermediate height at each experimental site (Takagi and Gyokusen, 2004) during the day from 09:00 to 14:00 HR on the sampling date. The shoot’s end was cut twice underwater to prevent cavitation and cut again just before taking measurements. Leaves were removed from the shoot and were analyzed on a leaf area meter (Li−3000, Li−Cor, Lincoln, NE, USA). Afterward, leaves were soaked in a 0.2 mM laboratory−grade calcium chloride solution for an hour to achieve water−saturated fresh weight, allowing calcium ions to bind to unsaturated cellular sites (Conway and Sams, 1987). Individual leaves were separately placed in aluminum foil, dried at 65°C until a constant dry mass was achieved, and then weighed. Specific leaf area (SLA, projected leaf area per dry mass) and leaf dry matter content (LDMC, projected water−saturated fresh weight per dry mass) were expressed to an international standard unit. The total number of trees listed in **Table 1** was used for the measurements.

### 2.4 Gas exchange measurements

Gas exchange characteristics, photosynthetic assimilation (*A*) and transpiration rates (*E*), and stomatal conductance (*g*_s_) were measured under controlled conditions using Li−6400 instrument equipped with 6 cm^2^ cuvettes and a 6400−02B red–blue light source (Li−Cor, Lincoln, NE, USA) on the sampling date during summer (July 17th to 27th, 2023) and autumn (September 22nd to October 6th, 2023). *A*/*E* was used to calculate instantaneous water use efficiency (WUE_i_). Leaves were prepared to measure gas exchange characteristics using a method similar to morphological traits. The shoot was then placed in a beaker filled with water and kept at room temperature at UIC’s Stable Isotope Laboratory. Gas exchange characteristics were measured with the following settings on the day of sampling: 1,200 µmol·m^−2^·s^−1^ photosynthetic photon flux density (PPFD), 1.9 ± 0.1 kPa vapor pressure deficit, relative humidity of 50–65%, constant leaf chamber temperature of 25°C, 400 µmol·mol^−1^ reference CO_2_ concentration, and a flow rate of 500 μmol·s^−1^. Gas exchange characteristics were measured when CO_2_ concentrations in the sample cell were stabilized, typically within 10 min. of exposure to 1,200 µmol·m^−2^·s^−1^ PPFD. Three leaves per tree were randomly selected on each sampling date (**Table 1**), and measurements were made between 10:00−16:00 HR.

### 2.5 Determination of carbon-to-nitrogen ratio and nitrogen content

Leaves were dried at 65°C, weighed, and ground using a SPEX 8000D Mixer Mill (SPEX SamplePrep LLC, Metuchen, NJ). The total carbon (C) and nitrogen (N) contents were analyzed using an elemental analyzer (Costech 4010, Milan, Italy), using a dry combustion method. The samples had organic C, no carbonates present, and the total N content included nitrate. The carbon-to-nitrogen (C/N) ratio, leaf N content per leaf area (NLA), and percentage of leaf N content (%N) were indicated. Two to four leaves were randomly selected per sampled tree at each site for the measurements.

### 2.6 Trait databases

The TRY database (TRY Plant Trait Database, 2023) and BIEN database ((BIEN) database, 2023) were utilized to obtain global data on tree leaf morphological and gas exchange responses. We qualitatively compared the global dataset and our dataset. See **Supplementary Table 1** for a complete list of the original publications associated with the subset of TRY and BIEN. Data from TRY and BIEN used here include various measurements of trees from two species (*Acer platanoides* and *Tilia cordata*) with measurements of leaf area, SLA, LDMC, *A*, *E*, *g*_s_, WUE_i_, C/N ratio, and NLA. Overlapping references in the TRY and BIEN databases are used only once. Some *A*, *E*, and *g*_s_ data were obtained from figures in publications extracted using the metaDigitise package in R (Pick et al., 2019). The extracted data records are uploaded in **Supplementary Table 1** for data archiving and sharing.

Our dataset for Norway Maple and Little−leaved Linden encompassed sample sizes ranging from 8 to 13 individuals at all the sites. In contrast, the sample sizes for morphological traits (leaf area, SLA, and LDMC) from the TRY, BIEN, and metaDigitised databases were substantially larger, ranging from 71 to 116 values for Norway Maple and 18 to 37 values for Little−leaved Linden. Additionally, the dataset for *A*, *E*, *g*_s_, and WUE_i_ from the databases contained 6 to 28 values and 9 to 37 values for C/N and NLA, covering both species.

### 2.7 Statistical analysis

Differences in T_a_−LST between two sites (urban park and suburban park) and LST between the urban park and both the suburban park and suburban residential site were compared using a paired Student’s *t*−test. SLA, LDMC, *A*, *E*, *g*_s_, and WUE_i_ were assessed between different sites (urban park, suburban park, and suburban residential sites) using a paired Student’s *t*−test (*p* < 0.05) to analyze the mean differences. One−way ANOVA with post−hoc Tukey Honestly Significant Differences (HSD) was performed to study the influence of site on leaf area, C/N ratio, NLA, and %N. The figures were drawn using OriginPro 2024 (OriginLab, Northampton, MA, USA). Gas exchange data were analyzed using linear regression models to evaluate the effects of site, season, and soil moisture on gas exchange parameters. All statistical analyses were conducted in R software (version 4.4.1).

## 3 Results

### 3.1 Temperature and soil moisture differences in urban park, suburban park, and suburban residential sites

The average daytime LST during the growing season in urban park was 0.9 to 1.6°C higher than in suburban park each year (**Table 2**). From 2018 to 2022, the daytime LST during the growing season in urban park, suburban park, and suburban residential sites were 21.9 ± 8.0 °C, 20.6 ± 7.4°C, and 21.4 ± 7.6°C, respectively. The average nighttime LST for the same period were 14.5°C, 13.8°C, and 13.9°C, respectively. Therefore, the LST difference between the urban park and both the suburban park and suburban residential site was significant (*p* < 0.001) (**Supplementary Figure 1**).

**Figure 2:**
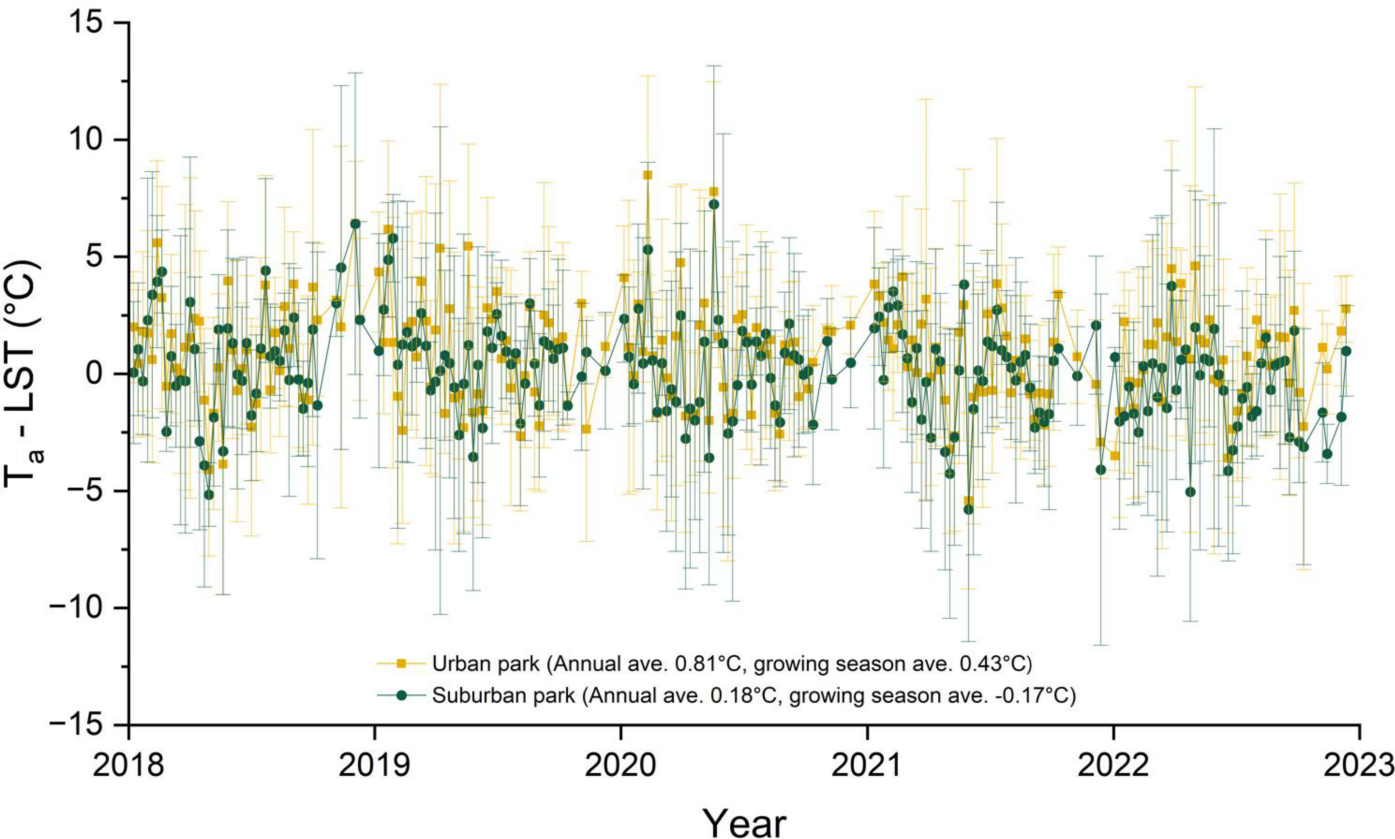
Weekly differences between air temperature (T_a_) and land surface temperature (LST) from 2018 to 2022. LST was measured at three locations: an urban park at 41°52’24.2”N 87°38’53.9”W (University of Illinois Chicago, Chicago, IL), a suburban park at 41°48’44.5”N 88°03’05.3”W (Lisle, IL), and a suburban residential site at 41°52’02.9”N 88°00’38.6”W (Lombard, IL). T_a_ data were obtained from the NOAA (2023) for the closest weather stations; Chicago Midway Airport (41°47’24.0”N, 87°44’24.0”W) for the urban park and Lisle Morton Arboretum Station (41°48’36.0”N, 88°03’36.0”W) for the suburban park. The annual and growing season (April to October) T_a_−LST differences were 0.81°C and 0.43°C for the urban park and 0.18°C and -0.17°C for the suburban park, respectively, with a statistically significant difference between the two (*p* < 0.001) based on the paired Student’s *t*−test. The plot displays weekly means with error bar representing ± standard deviation for each site.

**Figure 3:**
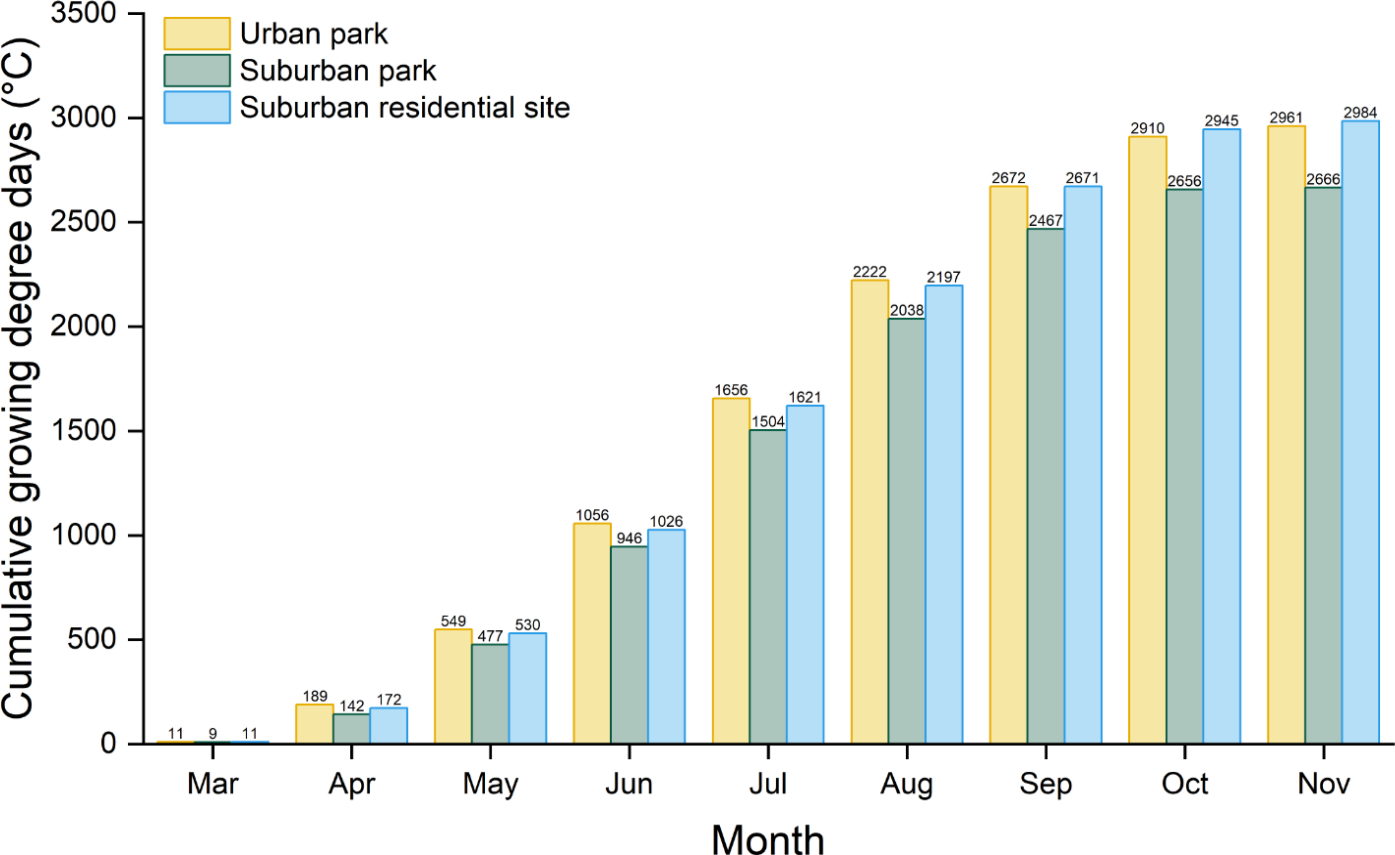
Accumulated monthly growing degree days (GDD) in 2023 for stations at three locations: the West Loop – Chicago Old St. Pats station at 41°52’48.0”N, 87°38’24.0”W for the urban park, Lisle Morton Arboretum Station at 41°48’36.0”N, 88°03’36.0”W for the suburban park, and the Downtown station in Lombard at 41°53’24.0”N, 88°01’12.0”W for the suburban residential site. The base temperature for calculating GDD is 5°C.

The urban park shows a substantial T_a_−LST difference compared to the suburban park, with annual and growing season averages of 0.81°C and 0.43°C, respectively, compared to 0.18°C and −0.17°C for the suburban park (**Figure 2**). This suggests greater heat retention in Chicago, IL, than in Lisle, IL. In 2023, cumulative GDD was highest in the urban park compared to the suburban sites during March and August. By September, cumulative GDD in the urban park and suburban residential sites were similar (2,672°C and 2671°C, respectively). However, throughout the growing season, cumulative GDD for both the urban park and suburban residential site were consistently higher than those for the suburban park (**Figure 3**).

Soil moisture during the summer was higher in the urban park (48.0−68.5%) compared to the suburban park (17.8-20.1%) and suburban residential (20.6−22.4%) sites for both Norway Maple and Little − leaved Linden (**Table 1**). By the fall, the soil moisture for Norway Maple was higher in the suburban park (29.5%) than in the urban park (16.3%) and suburban residential site (13.7%). For Little − leaved Linden, fall soil moisture was more consistent across all sites, with values ranging from 15.9% to 24.1%.

### 3.2 Differences in leaf functional traits across urban and suburban sites

#### 3.2.1 Leaf morphological traits

**Figure 4:**
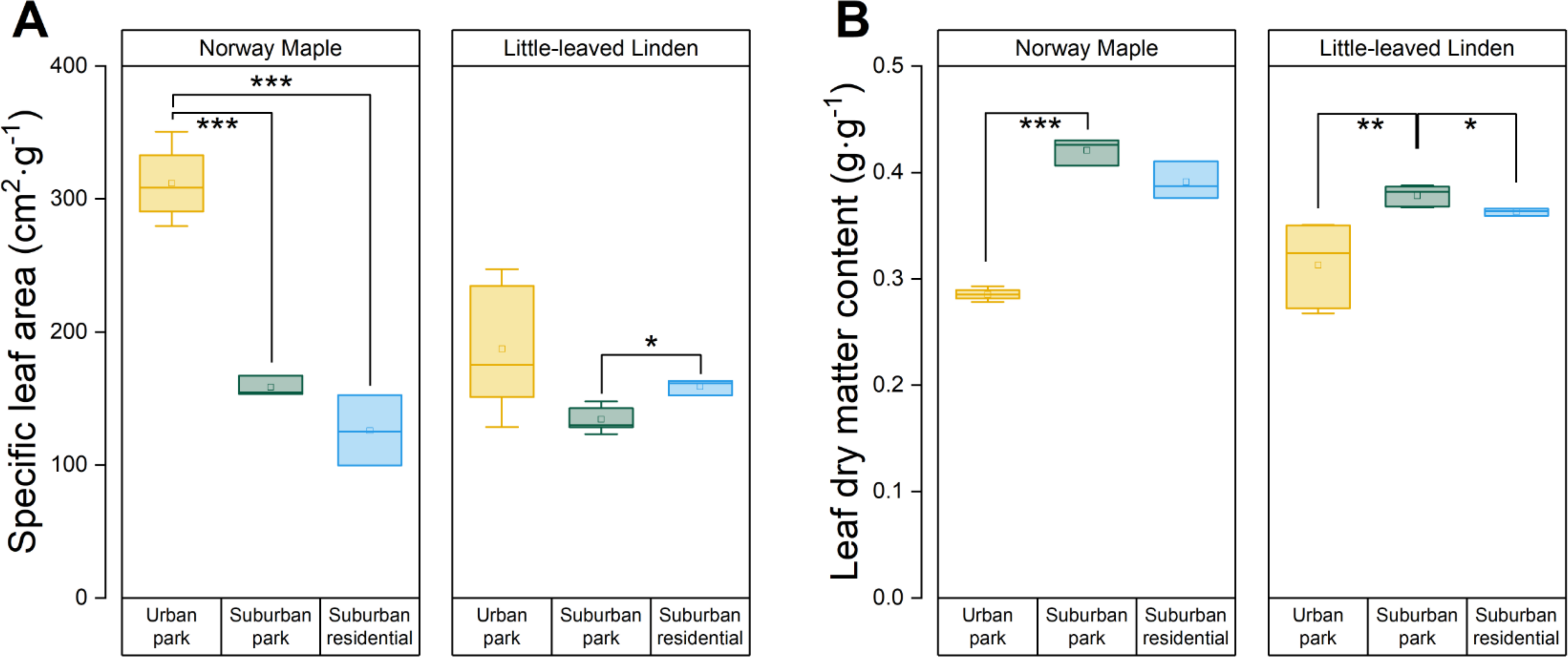
Analyses of LFTs of *Acer platanoides* (Norway Maple) and *Tilia cordata* (Little−leaved Linden) in urban park, suburban park, and suburban residential sites. **(A)** specific leaf area (SLA) and **(B)** leaf dry matter content (LDMC) was evaluated. LDMC was calculated by dividing the water−saturated fresh mass by the dry mass of leaves. Error bars represent 95% confidence intervals. Asterisks indicate statistically significant differences between the sites. Symbols *, **, and *** represent *p*−values of less than 0.05, 0.01, and 0.001, respectively, according to the paired Student’s *t*−test, while non−significant results (*p* > 0.05) were not indicated.

The SLA of Norway Maple was considerably greater in the urban park compared to suburban park site, but there was no significant difference between suburban park and suburban residential sites (**Figure 4A**). There was a large range of SLA for Little − leaved Linden in the urban park site, with values ranging from 129 to 247 cm^2^·g^−1^. Little − leaved Linden trees in the suburban residential site had a larger SLA than those in the suburban park site. Additionally, the LDMC of Norway Maple was lower in the urban park than in suburban sites (**Figure 4B**). Little − leaved linden exhibited a difference in LDMC between the urban and suburban park sites, with the former having a lower value. Although the average leaf area varied for both Norway Maple (76, 45, and 71 cm²) and Little − leaved Linden (43, 30, and 26 cm²) across the urban park, suburban park, and suburban residential sites, respectively, statistical analysis indicated no significant difference across sites for either species (**Table 3**).

**Table 3:** Leaf functional traits of *Acer platanoides* (Norway Maple) and *Tilia cordata* (Little−leaved Linden) in urban park, suburban park, and suburban residential sites.

Species	Site	Leaf area (cm^2^)	C/N ratio^z^	NLA (g·m^-2^)	%N
Norway Maple	Urban park	76.5 ± 3.5 a^y^	18.2 ± 1.0 b	0.66 ± 0.03 b	2.07 ± 0.11 a
	Suburban park	44.9 ± 6.8 a	31.2 ± 2.7 a	1.00 ± 0.10 b	1.48 ± 0.11 a
	Suburban residential site	70.5 ± 17.7 a	23.6 ± 1.8 ab	1.59 ± 0.15 a	1.92 ± 0.16 a
Little−leaved Linden	Urban park	43.1 ± 4.8 a	12.5 ± 0.4 b	1.69 ± 0.19 a	3.09 ± 0.13 a
	Suburban park	29.8 ± 8.3 a	17.0 ± 1.5 a	2.15 ± 0.15 a	2.66 ± 0.19 ab
	Suburban residential site	25.8 ± 2.8 a	14.7 ± 1.1 b	1.41 ± 0.06 a	2.18 ± 0.07 b

z C/N ratio = leaf carbon to nitrogen (C/N) ratio, NLA = leaf nitrogen content per leaf area, %N = percent of leaf nitrogen content.

y Values presented represent the average of two to four replications ± standard error. For differences among the sites from Norway Maple, ANOVA results are as follows: Leaf area, *F* = 2.79, *p* = 0.129; C/N ratio, *F* = 10.2, *p* = 0.017; NLA, *F* = 20.9, *p* = 0.004; %N, *F* = 3.58, *p* = 0.108. For differences among the sites from Little−leaved Linden, ANOVA results are as follows: Leaf area, *F* = 1.84, *p* = 0.208; C/N ratio, *F* = 6.18, *p* = 0.024; NLA, *F* = 3.72, *p* = 0.072; %N, *F* = 10.82, *p* = 0.05. Different letters (e.g., ‘a’ and ‘b’) indicate significant differences within each species and trait across sites based on Tukey’s range test at *p* < 0.05. The same letters indicate non−significant differences. Every variable showed the highest value for each species in the group “A”.

#### 3.2.2 Leaf gas exchange traits

**Figure 5:**
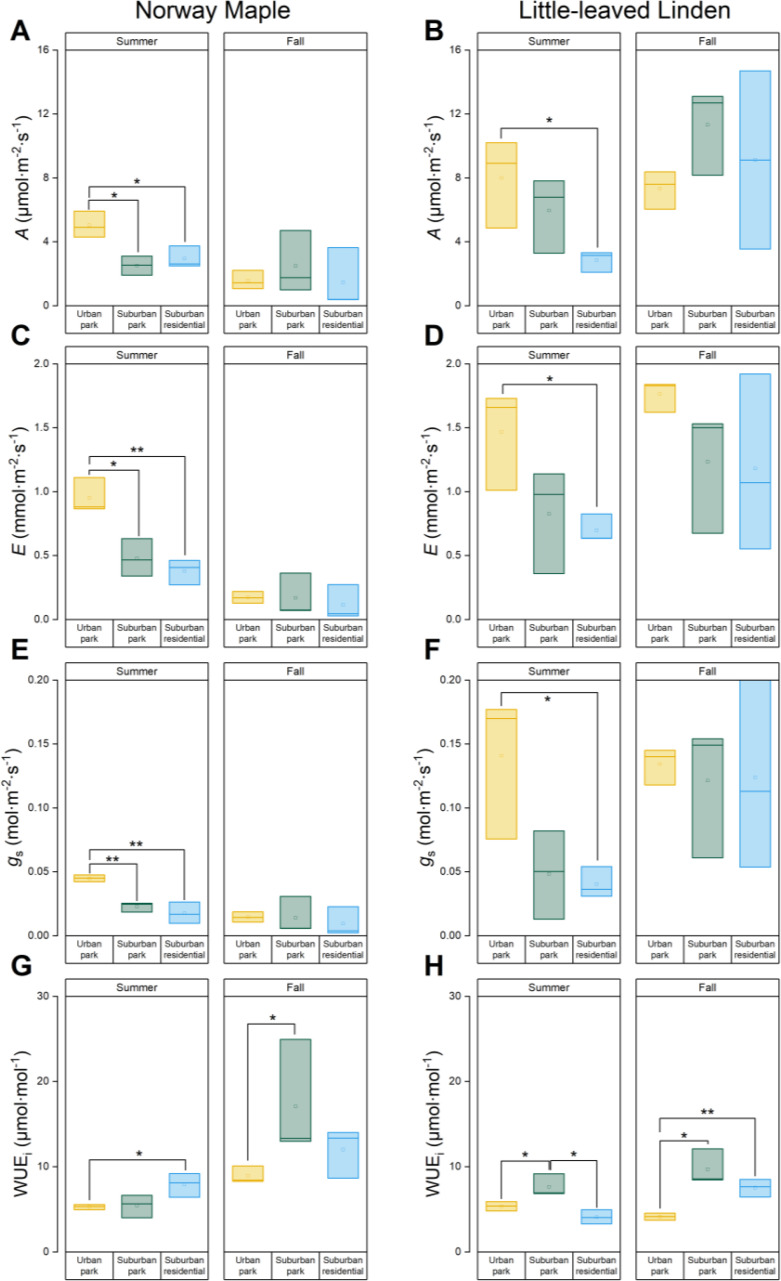
Analyses of gas exchange indexes of *Acer platanoides* (Norway Maple) and *Tilia cordata* (Little−leaved Linden) in urban park, suburban park, and suburban residential sites measured during summer **(A, C, E, G)** and fall 2023 **(B, D, F, H)**. **(A, B)** The photosynthetic assimilation rate *A*, **(C, D)** transpiration rate *E*, and **(E, F)** stomatal conductance (*g*_s_) were measured by a LI−6400 photosynthetic system. *A*/*E* was used to calculate **(G, H)** instantaneous water use efficiency (WUE_i_). Error bars represent 95% confidence intervals. Asterisks indicate statistically significant differences between the sites. Symbols * and ** represent *p*−values of less than 0.05 and 0.01, respectively, according to the paired Student’s *t*−test, while non−significant results (*p* > 0.05) were not indicated.

Norway maple trees had significantly higher levels of *A*, *E*, and *g*_s_ during summer in urban park compared to those in the suburban sites (**Figures 5A, C, E**). There was no significant difference between suburban park and suburban residential sites. Similarly, Little−leaved Linden trees had the highest *A*, *E*, and *g*_s_ values in urban park, which were significantly higher than those in the suburban residential site during the summer. However, *A*, *E*, and *g*_s_ did not differ significantly among different sites for Little − leaved Linden (urban vs. suburban) in fall (**Figures 5B, D, F**). Norway maple in the suburban residential site had higher WUE_i_ than those in urban park, and Little−leaved Linden had the highest WUE_i_ in suburban park between the sites during the summer (**Figures 5G, H**).

Norway Maple *A*, *E*, and *g*_s_ significantly decreased by 31, 18, and 32% in the urban park site from summer to fall, while WUEi increased by 69% (**Figures 5A, C, E, G**; **Supplementary Table 2**). Despite seasonal changes, all leaf gas exchange traits of Norway Maple remained relatively consistent in the suburban park and suburban residential sites, except for WUE_i_ in suburban park. *A*, *E*, and *g*_s_ of leaves in the urban park, suburban park, and suburban residential sites remained unchanged from summer to fall (**Figures 5B, D, F**; **Supplementary Table 2**). The WUE_i_ of Little−leaved Linden decreased in urban park and increased in suburban residential sites with seasonal changes, while remaining unchanged in suburban park (**Figure 5H**).

Taking into account the effects of site, season, and soil moisture, spring consistently influences gas exchange parameters, particularly by increasing *E* and decreasing WUE_i_ (**Supplementary Table 3**). Soil moisture generally exhibits weak or non-significant effects across both species, except for a significant positive effect on WUE_i_ in Norway Maple.

#### 3.2.3 Leaf elemental analysis traits

The leaf C/N ratio was significantly lower in trees located in the urban park compared to the suburban sites for both Norway Maple and Little−leaved Linden (**Table 3**). There was no significant difference in the leaf C/N ratio for both species between trees in the urban park and suburban residential sites. The %N was not significantly different between the sites for Norway Maple, but it was lower in the suburban residential site compared to the urban park site for Little−leaved Linden. For Norway Maple, the NLA was highest in the suburban residential site. The NLA did not differ significantly for Little − leaved Linden.

### 3.3 Qualitative traits comparison of the global database and our dataset

**Figure 6:**
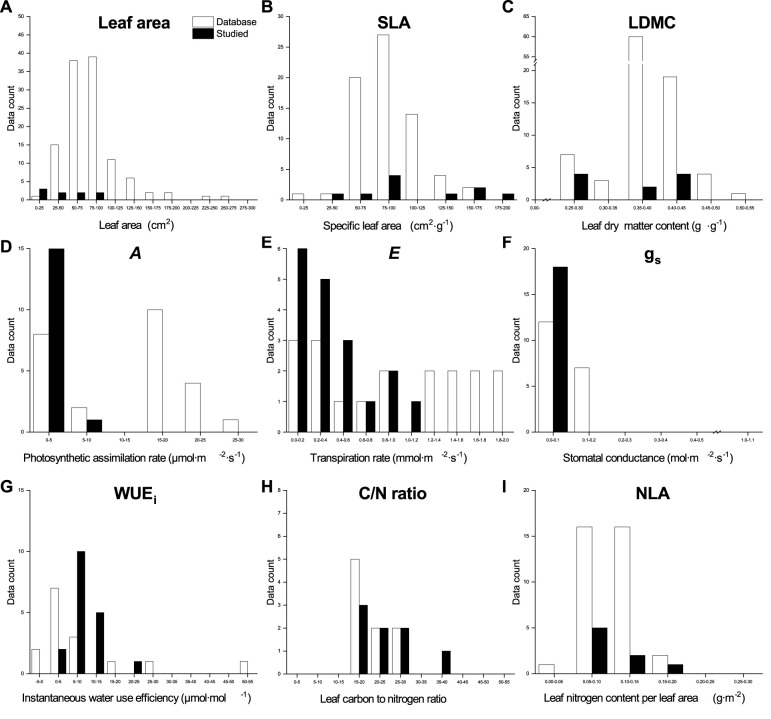
Qualitative comparison of the leaf functional traits database and our data for *Acer platanoides* (Norway Maple) in urban parks, suburban parks, and suburban residential sites. The database values were obtained from TRY and BIEN, and image processing using the R package metaDigitise in the literature listed in **Supplementary Table 1**.

**Figure 7:**
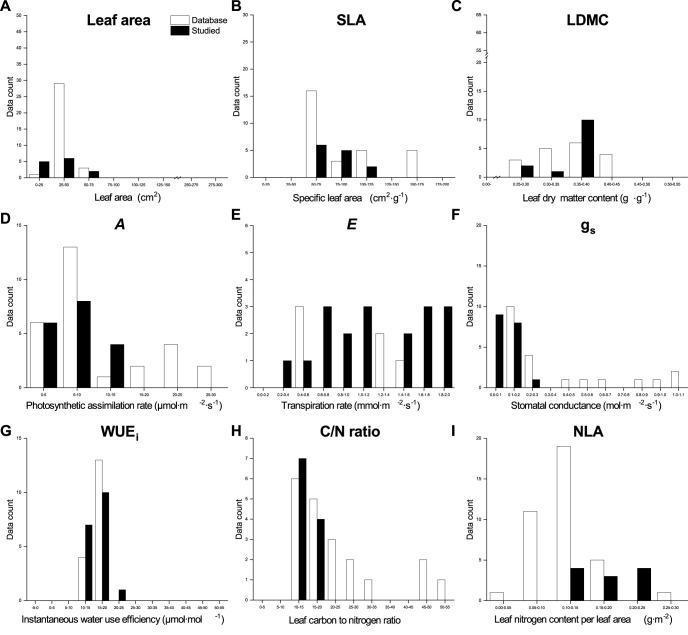
Qualitative comparison of the leaf functional traits database and our data from this study for *Tilia cordata* (Little − leaved Linden) in urban parks, suburban parks, and suburban residential sites. The database values were obtained from TRY and BIEN, and image processing using the R package metaDigitise in the literature listed in **Supplementary Table 1**.

The values for leaf morphological traits in our dataset for both species, except for the SLA of Norway Maple and leaf area of Little−leaved Linden, fell at the lower end of the range observed in the global databases (**Figures 6**, **7**). Both *A* and *g*_s_ values of both species in our dataset were at the lower end of the global databases’ range. However, our values of *E* and WUE_i_ for Little−leaved Linden were larger than those in the global databases. Elemental analysis values from our dataset for both species were compared to those in the global databases, indicating that C/N ratio values for Norway Maple in our dataset showed a wider distribution than those in the databases. The NLA values for Norway Maple in our dataset ranged from 0.58 to 1.80 g·m⁻², similar to those in global databases, which range from 0.47 to 1.80 g·m⁻². For Little − leaved Linden, our dataset’s C/N ratio was at the lower end, while the NLA was at the higher end of the ranges observed in the global databases.

## 4 Discussion

Trees exhibit phenological and morphological changes in response to environmental stressors in urban areas, such as to higher temperatures (Helletsgruber et al., 2020; Moser et al., 2016). Trees in the urban park site displayed superior cooling capabilities with higher SLA, lower LDMC, and increased gas exchange, indicating less evidence of water stress. Updating LFT databases with locally derived data is crucial as existing databases of LFTs may not fully capture the variability in urban environments.

Climate plays a significant role in shaping the life cycle of plants, leading to phenological shifts in response to global environmental changes (Vogel, 2022). Along the urban−suburban gradient, we observed a range of phenological drivers, with temperature emerging as a primary factor influencing morphological alterations (Su et al., 2023). The LST is typically lower in heavily forested areas due to the cooling effect from transpiration and shading provided by dense vegetation. Conversely, LST is higher in areas with a greater density of buildings and impervious surfaces as these urban features absorb and retain more heat, leading to the SUHI effect (Zhou et al., 2018). The LST analysis does not distinguish between general vegetation and tree cover for the urban park and suburban residential sites. This could affect the interpretation of vegetated areas and their impact on LST for those two sites. However, the high proportion of built−up area and low vegetated cover in urban Chicago may significantly amplify the difference between T_a_ and LST compared to the suburban Lisle area (**Table 2**; **Figure 2**).

The UHI effect not only adversely affects residents’ health and livability but also exacerbates stress and disrupts phenology in the limited vegetation present (Cregg and Dix, 2001; Zipper et al., 2016). Cumulative GDD in the urban park and suburban residential site, both with lower vegetation cover (22−23%), was significantly higher than in the suburban park, which had 35% tree cover and 24% vegetated area (**Figure 3**). This suggests that reduced vegetation cover may contribute to increased heat accumulation, as reflected in GDD values, highlighting the role of vegetation in moderating temperatures and mitigating urban heat. GDD has been increasing due to increased temperatures from global warming and can also be used to describe the UHI effect (Schatz and Kucharik, 2016; Kukal and Irmak, 2018). Many anticipated changes predicted by climate models in urban areas are already underway. Global urban stations mainly display positive UHI trends, with the nighttime trend peaking at 2.36 ± 0.69°C/century globally (Varquez and Kanda, 2018). In Chicago, heat waves are projected to intensify, increase in frequency, and prolong into the latter half of the 21st century (Meehl and Tebaldi, 2004). Larger vegetation cover plays a vital role in mitigating the impacts of high temperatures and other environmental stressors in urban areas by offering a variety of ecosystem services that counteract the adverse effects of climate change (Tan et al., 2021), especially in the Chicago Metropolitan Region.

Trees exhibit a heterogeneity of responses to environmental conditions along the leaf economic spectrum in urban areas, with quick or slow returns in investment (Wright et al., 2004). Species with quick returns in investment have leaves with high nutrient concentrations, high *A* and *g*_s_, short lifespan, and low LDMC (high SLA), while species with slow returns in investment have low leaf nutrient concentrations, low *A* and *g*_s_, long lifespan, and high LDMC (low SLA) (Poorter and Evans, 1998; Wright et al., 2004). Differences among and within species contribute to variations in trait responses as well as to unique environmental conditions (Fyllas et al., 2020). In addition to having higher SLA and lower LDMC, Norway Maple in the urban park site also displayed higher maximum rates of *A*, *E*, and *g*_s_ relative to the suburban sites during the summer (**Figures 4**, **5**). Higher temperatures stimulate photosynthesis in trees (Kanno et al., 2009; Sendall et al., 2015; Urban et al., 2017; Lahr et al., 2018). Urban trees growing in high-traffic areas demonstrated a 25% increase in SLA compared to those trees in areas with lower traffic levels (Gratani et al., 2000), in addition to temperature being a contributing factor. From 2017 to 2021, the three−year average of PM_2.5_ that have a negative impact on plant growth and yield was found to be higher at the Chicago sensing station located near Interstates (I)−90 and −94 compared to suburban sites such as Naperville, IL (IDPH, 2021). Our urban park trees grow in a highly urbanized area with high traffic and relative proximity to transportation corridors (I−290 and I−90) with exposure to high concentrations of air pollutants coupled with high temperatures. A study investigating the effects of particulate matter from incense on tree saplings showed that leaves of broadleaf species with higher SLA and *g*_s_ were more effective in reducing PM_2.5_ through gas exchange processes (Kim et al., 2022). Furthermore, the study revealed that leaves in urban parks had lower LDMC, indicating less solid material per unit area and more water content, similar to the findings from our study. This response could be an adaptation to higher temperatures in the city or higher water availability from flooding runoff or irrigation (Bijoor et al., 2012).

Water usage and availability play pivotal roles in urban tree adaptation strategies, particularly given the pronounced impact of elevated temperatures on evapotranspiration, tree growth, and overall sustainability (Lahr et al., 2018). Although soil moisture shows a weak relationship with most gas exchange variables in the linear regression model, it remains an important factor in understanding gas exchange dynamics, particularly for Norway Maple under higher soil moisture conditions at the urban site (**Table 1**; **Supplementary Table 3**). The higher soil moisture in the urban park could also be attributed to soil texture, which influences water movement and retention, as well as from other factors like irrigation, drainage from impermeable surfaces, and reduced competition for water compared to non-urban ecosystems (Halecki and Stachura, 2021). Soil moisture plays a critical role in alteration of leaf traits including SLA and gas exchange traits, and relative growth rate (Chaturvedi et al., 2013), even though other factors, such as temperature, may have a stronger impact based on the large impact of season for the gas exchange parameters.

Elevated rates of *A* and *g*_s_ in urban trees may suggest an ability to photosynthesize over a broader temperature range; however, this increase in gas exchange may come at the cost of lower WUE_i_, potentially making trees more susceptible to xylem embolism during high temperatures (Lahr et al., 2018). Although Norway Maple typically demonstrates isohydric behavior with stringent stomatal regulation, but it may exhibit anisohydric tendencies under certain environmental conditions (Leuschner et al., 2024). Norway Maple trees in the urban park site exhibited higher transpiration capabilities and thinner leaves compared to those in suburban sites, suggesting stronger cooling capacities both anatomically and physiologically, especially during the summer. Little−leaved Linden tends to adopt an anisohydric water−use strategy during drought periods, allowing for decreased leaf water potential (Moser et al., 2016). Throughout the summer and fall seasons, Little−leaved Linden in the urban park site maintained active gas exchange values, whereas these values declined in Norway Maple as fall approached. However, both species may face an elevated risk of hydraulic failure during periods of intense heat and drought, resulting in increased water loss and rapid soil moisture depletion (Rahman et al., 2019). Nevertheless, they remain suitable choices for tree planting in flood−prone and highly urbanized areas.

High SLA species invested more in thylakoid N per unit leaf mass and had a higher amount of Rubisco, indicating higher catalytic activity (Poorter and Evans, 1998). The C/N ratio was lower in the urban park site compared to the suburban park site in both Norway Maple and Little − leaved Linden, and the %N was higher in the urban park than in the suburban residential site, particularly for Little−leaved Linden that has smaller leaves than Norway Maple. According to Tang et al. (2016), nighttime warming promotes N uptake, which is associated with increased chlorophyll content and enhanced gas exchange traits in tree species. Assessing thylakoid N is necessary to understand metabolic N investment in trees; however, increased LST in urban environments can cause metabolic and phenotypic changes, leading to increased photosynthesis and transpiration.

We observed a strong influence of the sampling site and as well as the interaction between site and tree species on SLA, LDMC, leaf gas exchange traits, and elemental analysis traits, attributable to the urban−suburban environmental gradient (**Figures 6**, **7**). Although LFT values for both species vary across global databases, the range of values found in our study aligns closely with the global range, particularly for leaf area in Little−leaved Linden. We also observed that the leaf C/N of Norway Maple and *E* and WUE_i_ of Little−leaved Linden exceeded the ranges seen in the database. Plant communities experience ecological and evolutionary dynamics that regulate the cycling of C and nutrients among plant organs, shaping both plants and their environment (Kerkhoff et al., 2006). However, relationships between traits, such as SLA, can vary across communities due to climatic or edaphic factors, affecting the slopes and intercepts of key trait relationships (e.g. nutrient availability and plant growth). These variations reflect plant plasticity in response to environmental gradients, such as those observed at the urban-suburban scales, suggesting that global patterns of trait coordination may be disrupted by local heterogeneity and individual plastic responses (Freschet et al., 2013). Edaphic factors, such as soil texture and structure, play a critical role in determining soil moisture availability, which in turn influences tree functional traits and may contribute to the differences observed between sites (Lehmann and Stahr, 2007; Moser et al., 2016). It may be necessary to update the existing database of LFTs related to phenotypic plasticity in plants to incorporate more specific and localized responses to environmental changes that occur hierarchically (De Kroon et al., 2005). Coupling canopy−scale analyses with leaf−scale processes is essential for understanding the role of functional traits in shaping ecosystem structure and function (Abelleira Martínez et al., 2016). Furthermore, assessing genetic diversity is needed to determine whether phenotypic plasticity contributes to functional trait variation across urban and suburban environments (Gratani, 2014). Although ground measurements are time−consuming and limited in urban areas, they can provide valuable data for large−scale modeling efforts (Rissanen et al., 2024), aiding in a better understanding of the sources of variation in LFTs.

## 5 Conclusion

This study explored variations in morphological and physiological traits along an urban−suburban gradient in the Chicago Metropolitan Region. Results suggest that phenotypic variation in leaf traits was influenced by temperature, both nighttime and LST, as well as soil water content. Little − leaved Linden and, in particular, Norway Maple demonstrated significant differences in SLA, LDMC, *A*, *E*, and *g*_s_ along the gradient. Both species displayed anisohydric water−use strategies, taking advantage of urban irrigation despite the higher daytime temperatures in urban sites compared to suburban sites. Although both Norway Maple and Little − leaved Linden showed trends of having thinner leaves in urban sites, these differences were statistically significant only in Norway Maple. Little − leaved Linden in the suburban residential site exhibited similar trends to those observed in the suburban park, comparable to Norway Maple, potentially due to anthropogenic stressors influencing its physiological and morphological responses. Leaves from urban trees also demonstrated higher N investment, consistent with the increased *A* in leaves of urban trees, particularly in Little−leaved Linden. Across the urban−suburban gradient, variations in leaf traits exceeded the global range for certain traits (e.g., C/N ratio for Norway Maple and *E* and WUE_i_ for Little−leaved Linden), while other traits, such as SLA, LDMC, A, and g_s_ for both species exhibited skewed distributions compared to the global database. Taken together, these findings suggest that the most prevalent and abundant species in the Chicago Metropolitan Region can readily utilize water resources by expressing a wide variation in morphological traits, thereby maximizing the evaporative cooling capacity of urban trees. Furthermore, this study highlights the importance of using leaf trait characterization as a proxy for environmental factors, primarily water and temperature, in urban ecosystems.

## Data Availability

The original contributions presented in the study are included in the article/**Supplementary Material**. Further inquiries can be directed to the corresponding author.
