# Supplementary material for: Leaf functional traits highlight phenotypic variation of two tree species in the urban environment
Source: Front Plant Sci. 2024 Dec 17;15:1450723. doi: 10.3389/fpls.2024.1450723 (PMC11688637; doi:10.3389/fpls.2024.1450723)
Supplement: Supplementary file 1 [file DataSheet1.docx]

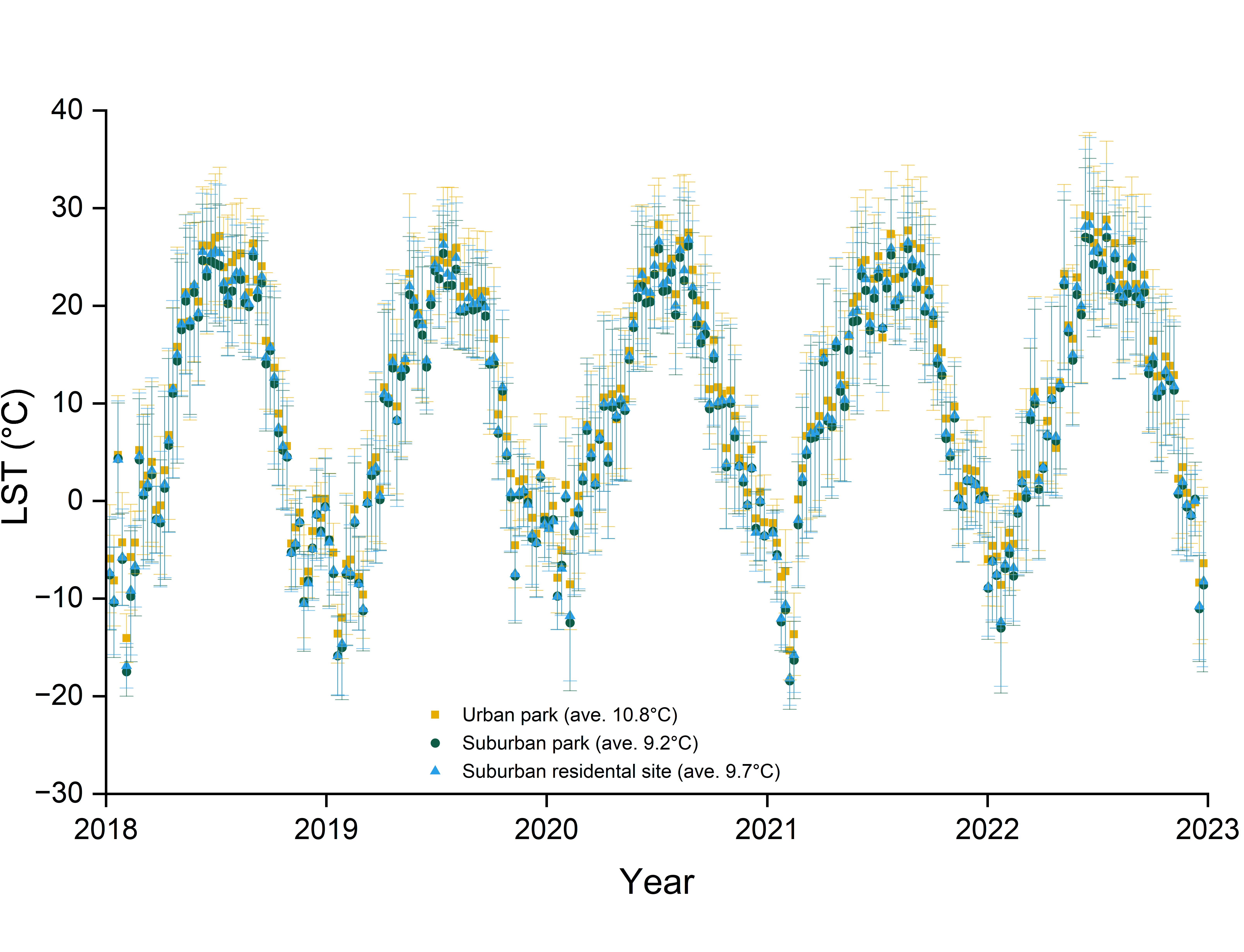


**Supplementary Figure 1.** Weekly land surface temperature (LST) from 2018 to 2022. LST was measured in three locations: an urban park at 41°52’24.2”N 87°38’53.9”W (University of Illinois Chicago, Chicago, IL), a suburban park at 41°48’44.5”N 88°03’05.3”W (Lisle, IL), and a suburban residential site at 41°52’02.9”N 88°00’38.6”W (Lombard, IL). The average LST was 10.8ºC for the urban park, 9.2ºC for the suburban park, and 9.7ºC for the suburban residential site, statistically significant differences observed between the urban park and both the suburban park and suburban residential site (*p* < 0.001) based on the paired Student’s *t*−test. The plot displays weekly means with error bar representing ± standard deviation for each site.

**Supplementary Table 2.** Significance *p*-value from the comparison of gas exchange variables between seasons (summer and fall) using a paired Student’s *t*−test.

| Species | Site | *A*^z^ | *E* | *g*_s_ | WUE_i_ |
| --- | --- | --- | --- | --- | --- |
| Norway Maple | Urban park | 0.004 | < 0.001 | < 0.001 | 0.004 |
|  | Suburban park | 0.981 | 0.074 | 0.369 | 0.044 |
|  | Suburban residential site | 0.269 | 0.052 | 0.379 | 0.093 |
| Little−leaved Linden | Urban park | 0.725 | 0.284 | 0.856 | 0.036 |
|  | Suburban park | 0.063 | 0.328 | 0.114 | 0.219 |
|  | Suburban residential site | 0.125 | 0.299 | 0.135 | 0.011 |

^z^*A* = photosynthetic assimilation rate, *E* = transpiration rate, *g*_s_ = stomatal conductance, WUE_i_ = instantaneous water use efficiency.

**Supplementary Table 3.** Coefficients from the linear regression model examine the effects of site (urban park and suburban residential site vs. suburban park), season, and soil moisture on gas exchange parameters. The table includes the estimated coefficients (Estimate), standard errors (S.E.), and *p*-values for each predictor. Significant predictors (p < 0.05) are noted.

| Species | Parameters^z^ | Variables^y^ | Estimate | S.E. | *p*-value^x^ |
| --- | --- | --- | --- | --- | --- |
| Norway Maple | *A* | Urban park | 0.070 | 0.793 | 0.931 |
|  |  | Suburban residential site | 0.486 | 0.785 | 0.546 |
|  |  | Season | 1.124 | 0.701 | 0.133 |
|  |  | Soil moisture^w^ | 0.054 | 0.033 | 0.123 |
|  | *E* | Urban park | -0.031 | 0.101 | 0.763 |
|  |  | Suburban residential site | 0.199 | 0.100 | 0.068 |
|  |  | Season | 0.384 | 0.089 | < 0.001*** |
|  |  | Soil moisture | 0.007 | 0.004 | 0.126 |
|  | *g*_s_ | Urban park | -0.003 | 0.006 | 0.590 |
|  |  | Suburban residential site | 0.009 | 0.006 | 0.119 |
|  |  | Season | 0.013 | 0.005 | 0.025* |
|  |  | Soil moisture | 0.0002 | 0.0002 | 0.351 |
|  | WUE_i_ | Urban park | 0.347 | 1.521 | 0.823 |
|  |  | Suburban residential site | -5.566 | 1.506 | 0.003 |
|  |  | Season | -8.883 | 1.346 | < 0.001*** |
|  |  | Soil moisture | 0.243 | 0.063 | 0.002 |
| Little−leaved Linden | *A* | Urban park | -2.343 | 1.962 | 0.254 |
|  |  | Suburban residential site | -1.839 | 2.225 | 0.424 |
|  |  | Season | -4.354 | 1.813 | 0.032* |
|  |  | Soil moisture | 0.053 | 0.070 | 0.457 |
|  | *E* | Urban park | -0.117 | 0.238 | 0.630 |
|  |  | Suburban residential site | 0.657 | 0.270 | 0.030* |
|  |  | Season | -0.337 | 0.220 | 0.149 |
|  |  | Soil moisture | -0.005 | 0.008 | 0.604 |
|  | *g*_s_ | Urban park | 0.001 | 0.030 | 0.962 |
|  |  | Suburban residential site | 0.041 | 0.033 | 0.242 |
|  |  | Season | -0.059 | 0.027 | 0.049* |
|  |  | Soil moisture | 0.001 | 0.001 | 0.506 |
|  | WUE_i_ | Urban park | -2.528 | 0.837 | 0.010* |
|  |  | Suburban residential site | -4.853 | 0.949 | < 0.001*** |
|  |  | Season | -2.180 | 0.774 | 0.015* |
|  |  | Soil moisture | 0.058 | 0.030 | 0.075 |

^z^*A* = photosynthetic assimilation rate, *E* = transpiration rate, *g*_s_ = stomatal conductance, WUE_i_ = instantaneous water use efficiency.

^y^Suburban park is the reference category for site comparisons in the model.

^x^Symbols * and *** represent *p*−values of less than 0.05 and 0.001, respectively

^w^The soil moisture is volumetric water content (%) in the top 12 cm of soil.
